# Coronary ostial stenosis after coronary artery bypass graft and combined aortic valve replacement: case report

**DOI:** 10.1097/MS9.0000000000000330

**Published:** 2023-03-27

**Authors:** Abdullah Nadeem, Wajeeha Bilal

**Affiliations:** Department of Medicine, Dow University of Health Sciences, Karachi, Pakistan

**Keywords:** angiography, angioplasty, aortic valve/surgery, CABG, cardiac surgical procedures/adverse effects, coronary artery, percutaneous coronary

## Abstract

A 65-year-old patient with the left anterior descending artery (LAD) ostial stenosis proved by coronary angiography is presented. LAD ostial stenosis is an uncommon condition whose etiology is unknown. The patient also had a coronary artery bypass graft combined with aortic valve replacement 13 years ago. The clinical and angiographic profiles of the patient are discussed here, supported by the literature.

**Case Presentation:**

A 65-year-old female patient with a medical history of hypertension and dyslipidemia came to the outpatient department with a complaint of chest pain and shortness of breath. Coronary angiography was carried out in 2008, which revealed triple vessel coronary artery disease, valvular heart disease, and ostial stenosis. In 2009, the patient underwent coronary artery bypass graft surgery combined with aortic valve replacement and remained asymptomatic thereafter. In 2022, transthoracic echocardiography and a Doppler study were conducted, which revealed normal size left ventricle, an ejection fraction of 55%, and diastolic dysfunction grade I. A graft study was done, which revealed left main and right coronary artery were normal, and the left circumflex artery with mild stenosis and obtuse marginal with subtotal stenosis and severe ostial stenosis of the LAD was observed.

**Clinical Discussion:**

Recognizing this complication early can prevent life-threatening complications and is then of the utmost importance. Coronary ostial stenosis is an uncommon but potentially dangerous consequence of aortic valve replacement whose etiology is not well understood in the literature. Rapid clinical identification is therefore essential. Coronary angiography needs to be done right away if coronary ostial stenosis is suspected. The mainstay of treatment for ostial stenosis is coronary artery bypass surgery or percutaneous coronary angioplasty. Since the patient has already undergone a coronary artery bypass graft (CABG) surgery, there is a significant risk of redoing CABG, as it is associated with considerable morbidity, which has a negative effect on long-term quality of life.

**Conclusion:**

Despite the fact that CABG is the most common form of therapy, percutaneous coronary intervention has demonstrated good short-term outcomes. To assess the effectiveness of CABG with drug-eluting stents for the treatment of coronary ostial stenosis, further information on long-term outcomes is required.

## Introduction

HIGHLIGHTS

- Coronary ostial stenosis is an uncommon but potentially dangerous consequence of aortic valve replacement.
- Unstable angina, left ventricular heart failure, ventricular arrhythmias, or sudden death are some of the symptoms of coronary ostial stenosis following aortic valve replacement.
- Despite the fact that coronary artery bypass grafting (CABG) is the most common form of therapy, percutaneous coronary intervention has demonstrated good short-term outcomes. Additionally, CABG is associated with considerable morbidity, which has a negative effect on long-term quality of life.
- To assess the effectiveness of CABG with drug-eluting stents for the treatment of coronary ostial stenosis, further information on long-term outcomes is required.

A rare but potentially dangerous consequence of aortic valve replacement (AVR) is coronary ostial stenosis. Roberts and Morrow provided the initial description of it in 1967^1^. It has been shown that patients receiving coronary artery bypass graft (CABG)-coupled AVR (CABG-AVR) may have a number of complications both before and after the procedure. Long-term complications for patients might include bleeding during or after surgery, blood clots, pneumonia, breathing issues, pancreatitis, renal failure, heart attack, stroke, lung issues, infection, irregular heart rhythms, graft failure, and death. Angiography is frequently used to diagnose coronary ostial stenosis rather than clinical examination. Although CABG remains the standard of care, individuals have also been effectively treated using percutaneous coronary intervention (PCI). Thus, if circulatory collapse or symptoms of myocardial ischemia happen quickly after surgery, it is crucial to have a high index of diagnostic suspicion. The SYNTAX score and the level of localization of the lesion serve as the primary determinants of therapy. Acute coronary syndrome, cardiogenic shock, and the number of arteries affected are further considerations. In this case report, we describe a patient who underwent CABG combined AVR for 13 years before developing ostial stenosis of the left anterior descending artery (LAD).

The reporting of the following case adhered to the Surgical CAse REport (SCARE) guidelines^2^.

## Timeline

**Table:** 

2008	The patient was diagnosed with class II angina according to CCS (Canadian Cardiovascular Society) grading. The patient was also diagnosed with ostial stenosis, coronary artery disease, and valvular heart disease
2009	The patient underwent coronary artery bypass graft surgery combined with aortic replacement. The patient recovered very well, and antiplatelet therapy was recommended for 12 months. The patient was then discharged from the hospital and remained asymptomatic
2013	Myocardial perfusion imaging was done using 8 mCi of Tc-99m isotope, which revealed normal findings. After this period, the patient remained asymptomatic upon examination in the outpatient clinic
2022	The patient presented with chest pain that was sharp, high in intensity, and radiates to the left arm, it worsens with exertion and was relieved by relaxation and medication. A graft study was done, which revealed left circumflex artery with mild stenosis and obtuse marginal with subtotal stenosis, and severe ostial stenosis of the LAD was observed in the proximal portion

## Case presentation

**Figure 1:**
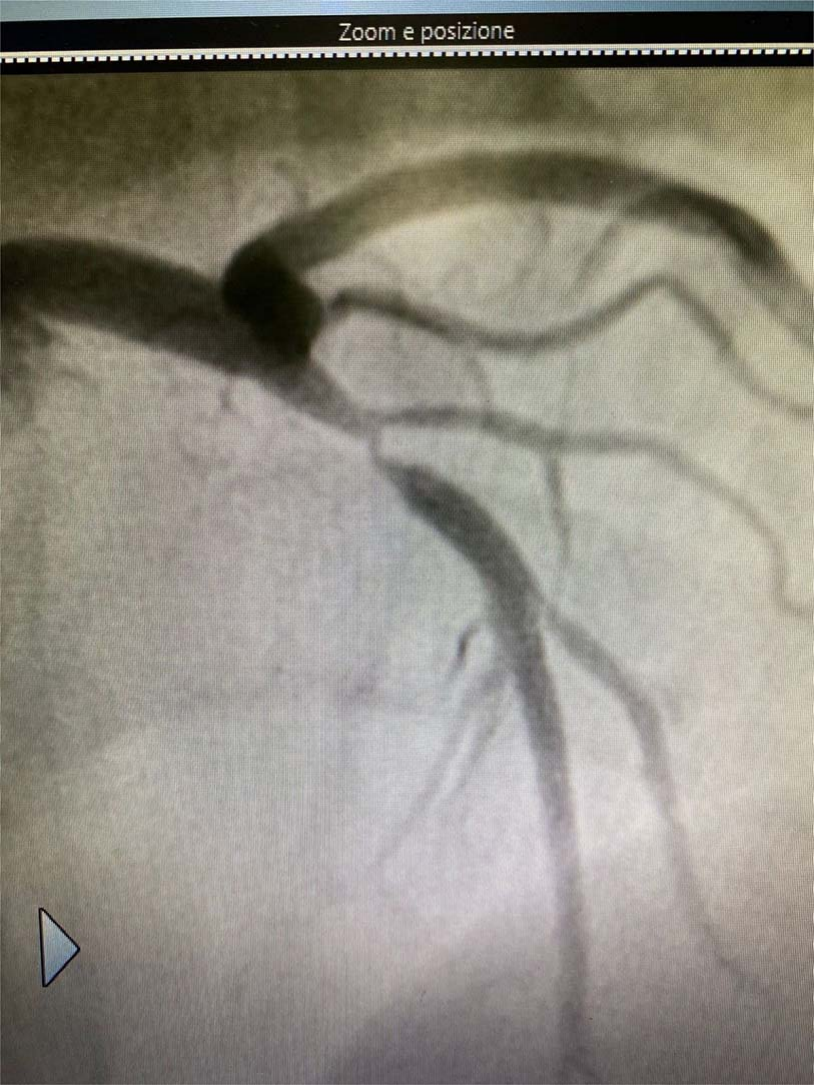
Coronary angiography of severe ostial stenosis of the left anterior descending artery.

**Figure 2:**
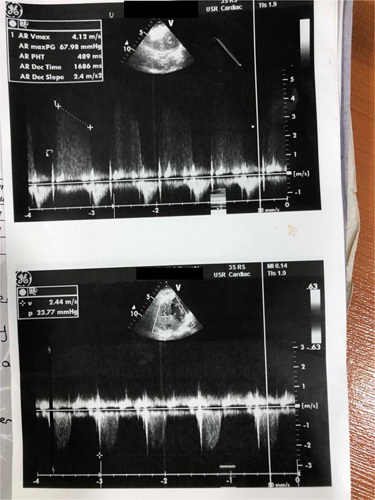
Echocardiogram of the patient. AR, aortic regurgitation; maxPG, maximum pressure gradient; PHT, pressure half-time.

In June 2022, a 65-year-old female patient with a history of hypertension and dyslipidemia came to the outpatient department with a complaint of chest pain and shortness of breath. According to the patient, she was in a normal state when suddenly she started having chest pain that was sharp, high in intensity, and radiated to the left arm, worsened with exertion, and was relieved by relaxation and medication. She also has shortness of breath. After taking history and examination, the patient was found to have paroxysmal nocturnal dyspnea and orthopnea. Her pulse was 68 and her blood pressure was 140/100. Past medical history revealed she had hypertension, cholelithiasis, and dyslipidemia. In 2008, she was diagnosed with class II angina according to CCS (Canadian Cardiovascular Society) grading. Coronary angiography was carried out in 2008, which revealed triple vessel coronary artery disease, valvular heart disease, and ostial stenosis, for which she underwent CABG-AVR in 2009. In CABG, the long saphenous vein was harvested from the right thigh and left leg. The pieces were joined to create an appropriate length. The left internal mammary artery was 1.5 mm, with excellent flow. LAD was found to have a diameter of 1.75 mm and was clean and located within the heart muscle (intramyocardial). The LAD was grafted with left internal mammary artery while the obtuse marginal was grafted with a reversed saphenous vein graft. In 2013, myocardial perfusion imaging was done using 8 mCi of Tc-99m isotope, which revealed normal findings. After this period, the patient remained asymptomatic upon examination in the outpatient clinic till June 2022. Transthoracic echocardiography and a Doppler study were done in June 2022, which revealed normal size left ventricle, an ejection fraction of 55%, and diastolic dysfunction grade I. A graft study was done, which revealed left main and right coronary artery were normal, the left circumflex artery with mild stenosis and obtuse marginal with subtotal stenosis and severe ostial stenosis of the LAD was observed in the proximal portion which can be seen in Figure 1. The patient was found to have ischemic heart disease with an increased risk of myocardial infarction. The patient is currently on drug therapy of risek, ascard, lipiget, flexiflow, monis, cancos, and sofvase. The echocardiogram of the patient is shown in Figure 2.

## Clinical discussion

Coronary ostial stenosis is an uncommon but potentially dangerous consequence of AVR and has been reported in previous studies^3^. Although coronary ostial stenosis following AVR can develop in the right coronary artery or the left main coronary artery, the disease is more common in the left coronary system^4^,^5^. The pathophysiology of coronary ostial stenosis has been explained by several different theories. One reason for coronary ostial stenosis is turbulent flow around prosthetic valves, which can lead to intimal thickening and fibrous proliferation close to the aortic root^6^,^7^. Furthermore, due to the infusion pressure of the cardioplegic fluid and the vessel’s over-dilatation, there is a risk of micro-injuries and localized hyperplastic responses^8^. Additionally, a bioprosthetic immunologic response is possible^9^.

In our case, the patient underwent an AVR along with a CABG 13 years earlier. A few years later, she started to experience symptoms such as dyspnea, chest discomfort, a protracted need for mechanical ventilation, and vascular and pulmonary problems. An angiography revealed ostial LAD stenosis, which explains the patient’s respiratory and cardiac issues. The angiography also revealed proximal LAD stenosis. Restenosis is more likely to occur in lesions in the distal or proximal arteries of the LAD, according to several studies^10^,^11^.

In general, unstable angina, left ventricular heart failure, ventricular arrhythmias, or sudden death are the symptoms of coronary ostial stenosis following AVR^12^. Rapid clinical identification is therefore essential. Coronary angiography needs to be done right away if coronary ostial stenosis is suspected^13^. While immediate coronary angiography is still the most accurate diagnostic method, transesophageal echocardiography can be helpful in the detection of acute complications after cardiac surgery. Despite the fact that CABG is usually the recommended course of action, PCI is a useful alternative because, in these circumstances, CABG may not always be a safe option due primarily to the close proximity of the graft site to the area of the prior operation^12^ as it can result in perioperative infarction, a high operative mortality rate, or a poor long-term outcome^14^. Additionally, CABG is associated with considerable morbidity, which has a negative effect on long-term quality of life^15^.

## Conclusion

Although it is uncommon, patients who arrive postoperatively with unstable angina, left ventricular heart failure, or life-threatening ventricular arrhythmias should be evaluated for coronary ostial stenosis. If there is a strong suspicion of coronary ostial stenosis, a comprehensive history and physical examination, as well as prompt coronary angiography, should be carried out. Due to possible acute coronary syndrome consequences, left ventricular heart failure, or ventricular arrhythmias, it may be fatal. Strategies for immediate reperfusion are essential in management. Despite the fact that CABG is the most common form of therapy, PCI has demonstrated good short-term outcomes. To assess the effectiveness of CABG with drug-eluting stents for the treatment of coronary ostial stenosis, further information on long-term outcomes is required.

## Ethics approval

This study was approved by the ethics committee of the institution.

## Patient consent

Written informed consent was obtained from the patient for the publication of this case report and accompanying images. A copy of the written consent is available for review by the Editor-in-Chief of this journal on request.

## Sources of funding

None.

## Author contribution

A.N.: study concept, collection of the data, drafting, literature review, data validation, supervision, and editing of the manuscript; W.B.: literature review and revising the manuscript for important intellectual content.

## Conflicts of interest disclosure

There are no conflicts of interest.

## Research registration unique identifying number (UIN)

This is not an original research project involving human participants in an interventional or an observational study but a case report; this registration was not required.

## Guarantor

Abdullah Nadeem.

## Data availability statement

All data underlying the results are available as part of the article and no additional source data are required.

## Provenance and peer review

Not commissioned, externally peer-reviewed.
